# Short dentin etching with universal adhesives: effect on bond strength and gingival margin adaptation

**DOI:** 10.1186/s12903-025-05490-9

**Published:** 2025-01-23

**Authors:** Hoda Saleh Ismail, Hanan Ahmed Nabil Soliman

**Affiliations:** 1 Conservative Dentistry Department, Faculty of Dentistry, Mansoura University, Algomhoria Street, Mansoura, PO Box 35516, Aldakhlia Egypt; 2 Conservative Department, Faculty of Dentistry, Kafr Al Sheikh University, Kafr Al Sheikh, Egypt

**Keywords:** Universal adhesives, Short dentin etching, HEMA-free adhesives, Bond strength

## Abstract

**Background:**

Short dentin etching, a relatively recent technique, aims to improve resin-dentin bonding by preserving hydroxyapatite crystals within the collagen spaces. This study explores short dentin etching’s potential in mitigating over-etching issues in deep proximal dentin/cementum margins, an aspect not previously investigated. This study evaluates the microtensile bond strength (μTBS) and marginal adaptation of two universal adhesives using different etch-and-rinse strategies (15-second and 3-second etching) and self-etch strategies, both immediate and post-thermal cycling and mechanical loading.

**Methods:**

Eighty-four molars underwent μTBS testing, categorized by the universal adhesive type (Tetric Uni and Prime&Bond Uni) (*n* = 42) and adhesive strategy (*n* = 14) with half tested after 24 h and the rest post aging. Forty-two molars received occluso-mesial preparations with proximal margins in dentin/cementum and were restored accordingly. Scanning electron microscope (SEM) examination of epoxy replicas for the restoration/gingival dentin interfaces was conducted after 24 h and aging. Dentin etching patterns were assessed using SEM. A three-way ANOVA evaluated μTBS data, while a two-way ANOVA and paired sample t-test analyzed marginal adaptation data (significance level is α = 0.05).

**Results:**

Adhesive type, strategy, and aging significantly influenced μTBS. After aging, Tetric Uni subgroups displayed higher bond strength compared to Prime&Bond Uni subgroups. Marginal adaptation was unaffected by adhesive type or strategy, although aging reduced bond strength and adaptation for both adhesives.

**Conclusions:**

The etch-and-rinse protocol yielded higher μTBS results for the HEMA-free isopropanol-based adhesive (Prime&Bond Uni). Marginal integrity was similar for both adhesives. The 3-second and 15-second etching times produced consistent results in all tests and for both adhesives.

**Clinical significance:**

The adhesive protocol for bonding universal adhesives to dentin is contingent on the adhesive composition.

## Introduction

The evolution of adhesive dentistry has spurred the development of versatile adhesives termed “universal,” “multimode,” or “multipurpose“ [1]. These adhesives offer application flexibility with a choice between self-etch and etch-and-rinse modes [2].

In a systematic review by Cuevas-Suárez et al. [3], mild universal adhesives showed consistent bonding performance to dentin across different strategies, indicating their suitability for a multimode approach. The optimal application mode (self-etch or etch-and-rinse) for effective dentin bonding remains debated [2, 4]. While past studies favored universal adhesives in self-etch mode for long-term in vitro performance [5], recent in vivo research has yielded conflicting results [6, 7]. Authors have highlighted the importance of functional monomers like methacryloyloxydecyl dihydrogen phosphate (10-MDP) in facilitating chemical bonding to dentin post-phosphoric acid application [8].

Universal adhesives have primarily been studied on non-etched or fully demineralized dentin [2], yet they present potential for resin-dentin bonding on selectively etched substrates, irrespective of dentin condition. Short dentin etching, a novel technique, aims to improve resin-dentin bonding by safeguarding hydroxyapatite crystals within deep dentin collagen spaces [9, 10]. This preservation can be achieved by using high-molecular-weight chelating agents or by reducing the acidity of traditional etchants [9, 11]. However, these chelating agents are less commonly available for clinical use compared to widely used 30–40% H_3_PO_4_ [9]. By reducing the etching time of H_3_PO_4_, higher calcium ratios in the hybrid layer can be maintained, potentially enhancing the stability of resin-dentin bonding with universal adhesives [2].

Short dentin etching, as evidenced in three studies [2, 12, 13], holds promise for enhancing bond strength. While two studies used a mild ethanol-based adhesive, which was previously reported in a systematic review to have comparable performance in both etch-and-rinse and self-etch methods [3], the third study focused on comparing universal adhesives with short dentin etching against their self-etch protocols, without referencing the standard 15-second etching time [13]. Further research is necessary to explore the potential of short dentin etching with different universal adhesives to improve retention rates in etch-and-rinse mode, thereby boosting bond strength and leveraging the chemical bonding advantages of these adhesives [3, 14].

Subgingival cavities below the CEJ present challenges in restorative dentistry. Bonding to etched enamel is effective, but dentin poses difficulties due to its organic composition, tubular structure, permeability, and lower surface energy [15]. The presence and thickness of cementum further complicate adhesion in these areas [15].

To address relocating the cervical margin above the CEJ, recommendations include using a traditional 3-step etch-and-rinse adhesive, simultaneous etching of thin interproximal enamel and dentin for a brief period, or employing 2-step self-etch adhesives without selective enamel etching [16, 17]. A recent systematic review suggests that bonding protocols and adhesive types do not significantly impact bond strength and marginal adaptation for deep subgingival margins [15]. Self-etch or universal adhesives in self-etch or selective enamel etch mode offer benefits for elevating deep margins to avoid over-etching dentin with etch-and-rinse adhesives [18]. The concept of short dentin etching may help address the issue of over-etching in these scenarios.

In this study, the aim was to assess the immediate and post-aging bond strength of two universal adhesives (HEMA-containing ethanol-based and HEMA-free isopropanol-based) using self-etch and two etch-and-rinse methods (15-second and 3-second etching). Marginal adaptation at dentin/cementum margins was also evaluated before and after aging, alongside dentin etching patterns observed through SEM. The study sought to test several null hypotheses: (1) The type of universal adhesive used would not affect bond strength or marginal adaptation under the same strategy and aging condition. (2) The adhesive strategy, rather than the duration of phosphoric acid etching, would not affect bond strength or marginal adaptation under the same aging condition. (3) Aging conditions would not affect bond strength or marginal adaptation under the same adhesive strategy. (4) No correlation would exist between bond strength data and marginal adaptation values in any subgroup.

## Materials and methods

### Materials used

This study aimed to investigate two mild universal adhesives with variations in monomer composition and solvent types. The adhesives under investigation were: (1) Tetric N Universal, HEMA-containing ethanol-based universal adhesive (Ivoclar Vivadent, Amherst, NY, USA), and (2) Prime&Bond Universal, HEMA-free isopropanol-based universal adhesive (Dentsply DeTrey GmbH, Konstanz, Germany). For detailed information on the materials used in the study, please refer to Table 1.

**Table 1:** Materials used in the study

Material	pH	Type	Manufacturer	Composition	Application technique	Lot number
Tetric *N*‑Bond Universal	2.5	HEMA-containing ethanol-based universal adhesive	Ivoclar Vivadent, Amherst, NY, USA	HEMA, Bis-GMA, D3MA, MDP, ethanol, water, methacrylate-modified polyacrylic acid, silicon dioxide, camphor quinone, ethyl *p*-dimethyl aminobenzoate, 2-dimethyl aminoethyl methacrylate.	**In self-etch mode:** 1. Apply bond with rubbing action for 20 s. 2. Disperse with oil- and moisture-free compressed air until a glossy, immobile film layer results 3. Light cure for 10 s. **In etch and rinse for either 3–15 s:** 1. Apply etchant for either 15–3 s. 2. Rinse thoroughly with a vigorous stream of water for the same period of etching and dry with oil- and water-free compressed air 3. Apply adhesive as in the self-etch strategy	Z04LY6
Prime&Bond Universal	2.5	HEMA-free isopropanol-based universal adhesive	Dentsply DeTrey GmbH, Konstan, Germany	Multifunctional acrylate, water, isopropanol, acidic acrylate, PENTA, 10-MDP, bifunctional acrylate, camphorquinone (photoinitiator), initiator, 4-(Dimethylamino) benzonitrile (photoaccelerator), stabilizer	**In self-etch mode:** 1. Apply a generous amount of adhesive to thoroughly wet all tooth surfaces 2. Agitate for 20 s. 3. Gently dry with clean air for at least 5 s. Surface should have a uniform, glossy appearance 4. Light-cure for 10 s **In etch and rinse for either 3–15 s:** 1. Apply etchant for either 15–3 s. 2. Rinse thoroughly for the same period of etching. 3. Remove rinsing water completely by blowing gently with an air syringe 4. Apply adhesive as in the self-etch strategy	2,205,000,297
TPH Spectra ST LV (A2)	Nanohybrid composite with pre-polymerized fillers		Dentsply DeTrey GmbH	**Fillers**: Spherical, pre-polymerized SphereTEC fillers, non-agglomerated fillers barium glass, and ytterbium fluoride. (Filler load: 76–78 wt%) **Resin matrix**: Urethane modified Bis-GMA resin; TEGDMA.		1136

Abbreviations: HEMA: hydroxyethyl methacrylate; Bis-GMA: bisphenol A-glycidyl methacrylate; D3MA: decandiol dimethacrylate; MDP: methacryloyloxydecyl dihydrogen phosphate; PENTA: dipentaerythritol penta acrylate monophosphate; TEGDMA: Triethylene glycol dimethacrylate

### Sample size calculation for μTBS test

The required sample size for μTBS testing was determined using GPower software (Ver. 3.1.9.7; GPower, Kiel, Germany). The calculation was based on a previous study with a similar design [2], considering the mean and standard deviation of self-etch and short dentin etching groups after aging (31.21 ± 6.87 and 42.97 ± 7.12, respectively). A two-tailed test with an effect size of 1.68, a significance level (α) of 0.05, 80% power, and an allocation ratio of 1 were considered. The calculated sample size per subgroup was 7.

### Sample size calculation for marginal adaptation test

The sample size calculation for the marginal adaptation test was based on a previous study that assessed the marginal adaptation of a universal adhesive used in both self-etch and etch-and-rinse modes with cervical margins of Class II cavities [19]. The mean and standard deviation of the gap were considered (15.79 ± 3.04 and 9.94 ± 2.78, respectively). A two-tailed test with an effect size of 2, a significance level (α) of 0.05, 80% power, and an allocation ratio of 1 were taken into account. The calculated sample size per subgroup was 6. An additional specimen was added to each group to accommodate the difference in study design.

### Microtensile bond strength testing

#### Selection and preparation of teeth

For this research, 84 human upper molars were selected for μTBS testing. These molars were extracted because of periodontal disease and were similar in size. They were carefully examined under a stereomicroscope (Olympus model SZ-PT, Tokyo, Japan) to ensure they were free of caries and cracks. Soft tissue and calculus were removed using an ultrasonic scaler, and the teeth were stored in a 0.5% Chloramine T solution. All teeth were used within six months of extraction. Written consent was obtained from the patients, and the Scientific Research Ethics Committee (KFSIRB200-260) approved the use of the teeth for research.

To streamline the process of preparing and restoring the teeth, the tooth roots were securely positioned vertically in cylindrical containers with an internal diameter of 29 mm and a height of 35 mm using a centralizing tool. Epoxy resin was poured into these cylinders, filling them up to 2 mm below CEJ. A specially designed jig device was used to ensure consistent and accurate positioning of each tooth during the fixation process.

To expose the mid-coronal dentin surfaces without damaging the pulp chamber, the occlusal surfaces of all teeth were cut parallel to the occlusal table and perpendicular to the long axis of the tooth. This was achieved by using a slow-speed diamond saw (Isomet 4000, Buehler Ltd., Lake Bluff, IL, USA) with coolant.

#### Experimental design and restorative procedures

After tooth preparation, the teeth were rinsed and dried. They were then randomly divided into two groups of 42 teeth each using simple randomization via Excel, based on the type of universal adhesive. Each group was further divided into three subgroups, each consisting of 14 teeth, based on the adhesive strategy: self-etch, etch and rinse with 37% phosphoric acid (N-Etch, Ivoclar Vivadent) applied for 15 s, and etch and rinse with a 3-second acid application.

To create a smear layer, the tooth surfaces were polished in a circular motion using 600-grit silicon carbide paper, ensuring a consistent and standardized smear layer formation with continuous water flow for 60 s. Care was taken to rinse and dry the dentin surfaces without excessive drying. The two universal adhesives were then applied to each subgroup, air-thinned and light-cured (LED curing light, Elipar Deep Cure; 3 M ESPE, St. Paul, MN, USA) with a power intensity of 1200 mW/cm^2^ following the manufacturer’s instructions (Table 1). For the etch and rinse specimens, after rinsing the etchant, the surfaces were air-dried for 10 s using an oil-free air flow three-way syringe, held at a 45-degree angle, and positioned approximately 1.5 cm away from the target area. The air pressure was set to 1 bar using a pressure regulator [20, 21].

In order to ensure consistency in building resin composite blocks, a custom-made Teflon mold with a rounded split design and a central square aperture (measuring 6 mm x 6 mm and 4 mm in height) was created. This Teflon mold was accurately positioned over the bonding surfaces using a specialized centralization tool [22]. A 4.0 mm-thick layer of nanohybrid resin composite (TPH Spectra ST LV, Dentsply DeTrey GmbH) was applied to restore the specimens. The composite material was applied in two 2 mm-thick horizontal increments using a gold-plated instrument (Zeffiro, Lascod SpA, Italy). Each increment was light-cured separately from the occlusal surface, according to the manufacturer’s recommendations. The curing process was monitored using a radiometer (Demetron L.E.D. Radiometer, Kerr Corp., Orange, CA, USA) after every five specimens. To achieve a smooth surface and improved adaptation of resin composite, a clear polyester Mylar strip, 10 mm wide, was applied to the top layer. A transparent glass slide and a 500-gram weight were then placed on the strip for half a minute. After this period, the weight and glass slide were removed, and the surface was cured by pressing a light tip closely against the polyester strip.

After removing the Teflon mold, an additional round of light curing was performed for 20 s on all restorations from the side. The specimens were then stored in distilled water at 37 ± 1 °C in an incubator for 24 h. All tooth preparation and restoration procedures were conducted by a single operator throughout the study using magnifying loupes (×4 loupes, Amtech, Wenzhou, China) and LED headlight illumination (HLP05, Amtech).

#### Artificial aging

In each subgroup of adhesive strategies, specimens were randomly allocated to two distinct aging conditions, with seven specimens assigned to each condition. The initial condition involved immediate testing following a 24-hour incubation in sterile water at 37 ± 1 °C. The second condition included both thermal cycling and mechanical loading procedures.

Thermal cycling was performed using an SD Mechatronik Thermocycler from Germany, subjecting the specimens to 10,000 cycles to replicate a year of clinical service, in accordance with ISO 11,405 guidelines [23, 24]. The cycling temperatures ranged between 5 °C and 55 °C (within a ± 2 °C tolerance range), with a 25-second dwell time and a 5-second transfer interval between baths [25].

Mechanical loading was carried out using a four-station multi-modal ROBOTA chewing simulator (ROBOTA Model ACH-09075DC-T, Ltd., AD-Tech Technology Co., Germany) operated by a servo motor. A force equivalent to 5 kg, corresponding to 49 N of chewing force, was applied. This testing regime was repeated 150,000 times to simulate one year of clinical chewing conditions, as recommended by a previous systematic review [26]. Thermal cycling preceded mechanical loading in the testing sequence [27].

**Fig. 1:**
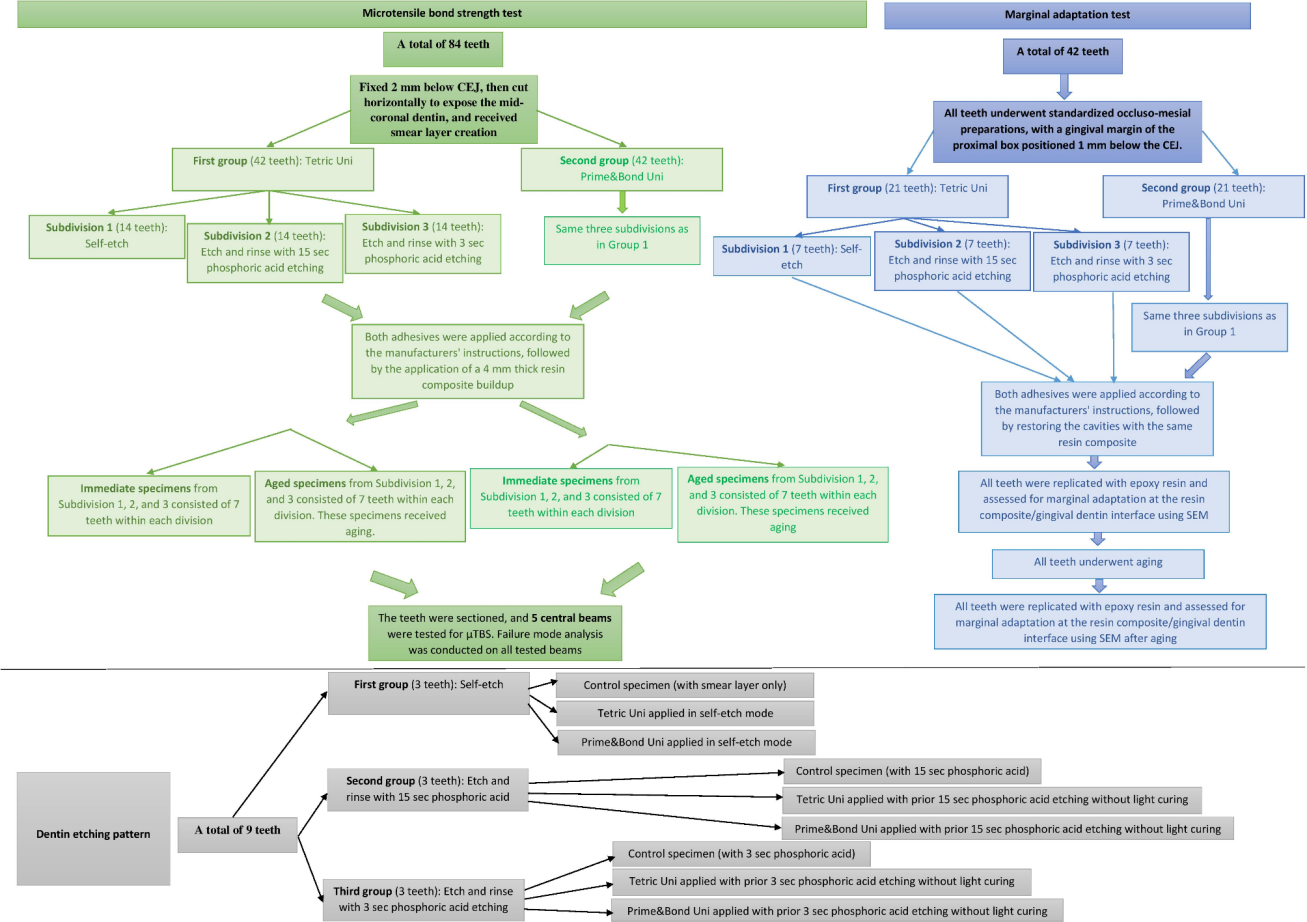
A schematic illustration depicting the experimental grouping and all the steps involved in specimen preparation for the microtensile bond strength, marginal adaptation tests, and dentin etching pattern evaluation in the current study

Post-aging, all specimens were examined for damage under an optical microscope. Each tooth within a subgroup was identified by a specific color and numbered 1 to 7, with the central area of the resin composites marked before sectioning for testing. A schematic illustration of the experimental grouping and all the steps involved in specimen preparation for the μTBS test is presented in Fig. 1.

#### Specimen preparation

Specimens were prepared as rectangular beams by cutting them perpendicular to the bonded interface with a slow-speed diamond saw and water coolant. Each beam had a cross-sectional area of 1 mm², comprising resin composite on top and coronal dentin on the bottom. Dimensions were precisely measured using a digital caliper with 0.01 mm accuracy. Five central beams were randomly selected from each specimen for testing.

For the microtensile bond strength evaluation, the beams were secured in Geraldeli’s jig and attached to an Instron universal testing machine (Model: 3345, Norwood, MA, USA). They were fixed in place with cyanoacrylate-based glue (Zapit, DVA Inc, Corona, CA, USA) and connected to the machine via a 500 N load cell. A tensile load was gradually applied at a cross-head speed of 0.5 mm/minute until the beams failed. The bond strength was calculated in MPa using Bluehill Lite software (Instron, Norwood). After testing, the fragments were removed from the jig and inspected under a stereomicroscope (Olympus model SZ-PT) at 40× magnification to identify the failure mode, which could be adhesive, cohesive within the resin or dentin, or mixed. Specimens that failed before testing were documented but excluded from further statistical analysis. All test procedures were carried out by a skilled operator who was unaware of the restorative steps.

### Marginal adaptation

#### Teeth selection, fixation, and Cavity Preparation procedures

Forty-two upper molars were selected and fixed as described in the μTBS study. A standardized set of occluso-mesial preparations was performed using a medium-grit diamond bur and a high-speed handpiece with water coolant. The preparations had consistent dimensions: a bucco-lingual width of 3 mm and an occlusal depth of 3 mm measured from the cavosurface margin of the cavity. For the box part, the base had a mesio-distal dimension of 1.5 mm, a bucco-lingual width of 3 mm, and extended 1 mm below the CEJ [28]. Accurate measurements were obtained using a graduated periodontal probe. After the preparation, a thorough examination of the cavities was conducted.

The teeth were randomly assigned to two groups (*n* = 21) based on the type of universal adhesive used. Within each group, the teeth were further divided into three subgroups (*n* = 7) based on the adhesive strategy for the bond strength test. Each subgroup’s teeth were marked with specific colors and sequentially numbered from 1 to 7.

#### Restorative procedures

After preparing the cavities, selective etching was done on the occlusal and proximal enamel margins using 37% phosphoric acid for 15 s, followed by rinsing and drying. In the dentin acid-etched subgroups of each universal adhesive group, the proximal gingival dentin margins were etched with phosphoric acid for either 15–3 s, followed by rinsing and drying. The universal adhesive was applied to all cavity surfaces, air-thinned, and light-cured according to the manufacturer’s instructions.

To ensure proper sealing, Tofflemire retainers and metal matrix bands were placed around each tooth, extending beyond the gingival margin of the cavity. An Ivory matrix holder no. 1 with a rubber piece on each prong of the retainer was securely fastened over the mid-mesial and mid-distal surfaces, pressing the Tofflemire matrix-band against the two proximal surfaces of each tooth. Visual and tactile inspection with magnification and an explorer confirmed a complete seal at the gingival margins. Subsequently, all teeth were restored using the same resin composite material used in the bond strength test. The composite was inserted into the cavity in three 2 mm-thick horizontal increments and cured for 20 s from the occlusal surface. After removing the matrix-band, an additional 20-second curing was performed from the proximal surface.

Finishing and polishing were carried out using Al_2_O_3_ discs (Extra-Thin Sof-Lex discs, 3 M ESPE) and a low-speed handpiece with water cooling. The specimens were then subjected to ultrasonic cleaning after being removed from their fixation blocks. It is important to note that a single operator performed all the preparation and restoration procedures using magnification. A schematic illustration of the experimental grouping and all the steps involved in specimen preparation for the marginal adaptation test is presented in Fig. 1.

#### Marginal adaptation evaluation using SEM

For a detailed protocol regarding the recording of restoration margins, SEM evaluation, and scoring, please refer to another study [28]. In summary, the mesial surfaces of all teeth were cleaned, and addition silicone impression materials were utilized to make impressions. These impressions were allowed to polymerize for 12 h and then filled with epoxy resin. The replicas were air-dried for 24 h at room temperature, mounted on aluminum stubs, and coated with a layer of gold using a sputter-coater.

To examine the restoration/gingival margin interface, a SEM (JSM-6510LV, JEOL Ltd., Tokyo, Japan) was employed at a magnification of 30× to obtain an overall proximal view. Image analysis software was used to analyze and measure each section of the restoration/gingival dentin interface at a magnification of 200×. The marginal integrity of each restoration and gingival dentin was evaluated by determining the percentage of continuous margin (% CM), which represented the length of the perfectly sealed margin relative to the total length of both perfect and imperfect margins, measured in micrometers. Margins were classified as either continuous/gap-free or discontinuous/gap based on a predefined protocol [28]. All SEM examinations and measurements were conducted by a single operator who was unaware of the restorative procedures. The intraexaminer reliability of the measurements was assessed by having the same examiner repeat the measurement procedures after a two-week interval, using the intraclass correlation coefficient (ICC).

#### Artificial aging

Following the initial assessment of the margins, all teeth underwent thermal cycling and mechanical loading according to the specific parameters outlined in detail in the bond strength section.

#### Evaluation of marginal adaptation after aging

Following the artificial aging procedure, the restoration/gingival dentin interfaces were reevaluated to assess their marginal adaptation. The same techniques and criteria used in the initial pre-aging evaluations were applied.

### Dentin etching patterns

This test utilized a total of nine teeth, which underwent fixation and cutting until reaching the mid-coronal dentin, as described in the bond strength section. The teeth were trimmed 2 mm below the CEJ. Following this, the root portion of each section was embedded in epoxy resin blocks measuring 5 mm in height for easier manipulation, ensuring that the mid-coronal dentin surface faced upwards. A smear layer was formed on all dentin surfaces. The nine dentin sections were then randomly divided into three groups based on the three adhesive strategies employed. Further subdivisions were made within each group based on the universal adhesive used (*n* = 1 for each). Additionally, one specimen within each group was designated as a control (without adhesive application) (Fig. 1).

Group 1: Occlusal surfaces were either untreated (control) or treated with universal adhesives (Self-etch strategy) without curing.

Group 2: One dentin disc was etched for 15 s with phosphoric acid, while the other two received universal adhesive without curing after the same etching duration.

Group 3: One disc was etched for 3 s with phosphoric acid, and the other two were treated with universal adhesive without curing after the same etching duration.

The resin monomers were rinsed off, and then dehydrated using a series of ascending ethanol concentrations (50%, 70%, 80%, 90%, and 3 × 100%) [12]. Specimens were mounted, coated, and analyzed using SEM at 2,000× and 5,000× magnifications.

### Statistical analysis

Bond strength values (MPa) were calculated as the mean μTBS of five beams per tooth. SPSS software (version 20) was used for statistical analysis, which revealed a normal distribution of μTBS values, allowing for parametric tests. A three-way ANOVA assessed the effects of universal adhesive type, adhesive strategy, and aging condition on bond strength, with post-hoc analysis using the Bonferroni adjustment (α = 0.05). Cross-tabulations and the Chi-Square test were used to analyze the distribution of failure types. Pre-test failure data were analyzed using independent t-tests for universal adhesive type and aging condition, and a one-way ANOVA for adhesive strategy. The ICC was used to evaluate the examiner’s measurement reliability for %CM data. A two-way ANOVA analyzed the effects of universal adhesive type, adhesive strategy, and their interactions on %CM values within each aging condition. Paired-sample t-tests examined the effect of aging on %CM values for each restorative system, as the difference between paired groups was normally distributed (α = 0.05). Pearson’s correlation coefficient assessed the correlation between μTBS and %CM values.

## Results

### μTBS results

Table 2 presents the mean μTBS values, standard deviations, and coefficients of variation for all the examined subgroups. A three-way ANOVA confirmed significant effects of all variables on bond strength values (adhesive type: *p* < 0.001, adhesive strategy: *p* = 0.04, and aging: *p* < 0.001). All interactions between variables were insignificant except for the interaction between adhesive type and adhesive strategy (adhesive type and adhesive strategy: *p* = 0.007, adhesive type and aging: *p* = 0.852, adhesive strategy and aging: *p* = 0.986, adhesive type, adhesive strategy, and aging: *p* = 0.938). The coefficient of variation varied across the subgroups, ranging from 14.18 to 25.2%.

**Table 2:** Mean ± SD (coefficient of variation within the subgroup) of microtensile bond strength values in MPa calculated for tested universal adhesives using different adhesive strategies, both immediately and after aging

Tested universal adhesive	Adhesive strategy	Immediate	Aged
Tetric Uni	SE	39.43 ± 7.13 (20.75)^a^	30.08 ± 4.25 (14.18)^ab^
	ER 15s	36.89 ± 5.78 (15.57)^a^	28.4 ± 4.17 (14.59)^b^
	ER 3s	38.57 ± 7.22 (18.76)^a^	29.9 ± 5.68 (18.97)^b^
Prime&Bond Uni	SE	27.06 ± 4.93 (18.38)^bc^	19.09 ± 4.82 (25.20)^d^
	ER 15s	34.17 ± 7.23 (21.14)^a^	25.03 ± 4.74 (19.01)^cd^
	ER 3s	35.11 ± 5.93 (16.92)^a^	27.17 ± 4.65 (17.01)^c^

Abbreviations: SE: Self-etch; ER 15s, Etch and rinse with 15 s phosphoric acid etching; ER 3s, Etch and rinse with 3 s phosphoric acid etching. The statistical differences were determined through a three-way ANOVA with post-hoc analysis employing the Bonferroni adjustment (α = 0.05). Groups identified with the same superscripted lower case letters are not significantly different from each other

In comparing the μTBS values of the two universal adhesives under the same adhesive strategy immediately, no significant differences were found, except for the Prime&Bond Uni self-etch subgroup. This subgroup exhibited significantly lower bond strength compared to the other adhesive strategy subgroups within the same adhesive category, as well as all immediate subgroups of Tetric Uni adhesive (*p* < 0.05).

After aging, the Tetric Uni subgroups, which showed no significant differences among themselves, exhibited significantly higher bond strength compared to all Prime&Bond Uni subgroups. Among the Prime&Bond Uni subgroups after aging, the etch and rinse strategy subgroups had higher bond strength compared to the self-etch strategy subgroups, with statistical significance observed in the etch and rinse 3s subgroup. It is clear that aging had a detrimental effect on all universal adhesives tested, regardless of the adhesive strategy used.

### Failure patterns

Table 3 illustrates the distribution of failure modes and pre-test failures across all subgroups, presented as percentages. A notable interaction was observed between failure patterns and aging (*p* = 0.006). However, no significant interactions were found between failure patterns and adhesive type or adhesive strategy (*p* = 0.832, *p* = 0.706). Regardless of the subgroup analyzed, adhesive failure patterns predominated, followed by mixed patterns. After aging, adhesive failures increased, while cohesive failures decreased. Pre-test failure was analyzed in relation to adhesive type and aging condition using an independent t-test, but no significant differences were found (*p* = 0.36, *p* = 0.46, respectively). Additionally, a one-way ANOVA revealed no statistically significant variation in pre-test failure rates with regard to adhesive strategy (*p* = 0.16).

**Table 3:** Failure modes distribution and pre-test failure in percentages (number of beams) for tested universal adhesives using different adhesive strategies, both immediately and after aging

Tested universal adhesive	Adhesive strategy	Immediate					Aged				
		Adhesive	Cohesive in composite	Cohesive in dentin	Mixed	Pre-test failure	Adhesive	Cohesive in composite	Cohesive in dentin	Mixed	Pre-test failure
Tetric Uni	SE	40% (14)	11.4% (4)	5.7% (2)	34.3% (12)	8.6% (3)	57.1% (20)	5.7% (2)	0% (0)	25.7% (9)	11.4% (4)
	ER 15s	42.9% (15)	17.1% (6)	8.6% (3)	22.9% (8)	8.6% (3)	37.1% (13)	8.6% (3)	2.9% (1)	45.7% (16)	5.7% (2)
	ER 3s	42.9% (15)	20% (7)	5.7% (2)	22.9% (8)	8.6% (3)	54.3% (19)	8.6% (3)	0% (0)	31.4% (11)	5.7% (2)
Prime&Bond Uni	SE	40% (14)	8.6% (3)	11.4% (4)	28.6% (10)	11.4% (4)	57.1% (20)	8.6% (3)	2.9% (1)	22.9% (8)	8.6% (3)
	ER 15s	37.1% (13)	14.3% (5)	0% (0)	42.9% (15)	5.7% (2)	62.9% (22)	5.7% (2)	0% (0)	22.9% (8)	8.6% (3)
	ER 3s	42.9% (15)	14.3% (5)	0% (0)	31.4% (11)	11.4% (4)	45.7% (16)	5.7% (2)	0% (0)	40% (14)	8.6% (3)

Abbreviations: SE: Self-etch; ER 15s, Etch and rinse with 15 s phosphoric acid etching; ER 3s, Etch and rinse with 3 s phosphoric acid etching

### Marginal adaptation results

The level of agreement between the two sets of % CM data measurements was high, as indicated by an ICC value of 0.96, suggesting strong consistency within the examiner. Therefore, the average of both sets was used for further analysis.

Table 4 presents the average % CM values and standard deviations for the different universal adhesives tested using various adhesive strategies, both immediately and after aging. Statistical analysis using a two-way ANOVA showed that neither adhesive type nor adhesive strategy had a significant effect on the results (*p* = 0.482 and *p* = 0.312, respectively). Moreover, no statistically significant interaction between these variables was found (*p* = 0.766).

**Table 4:** Mean ± SD (%) of % CM values of marginal adaptation for tested universal adhesives using different adhesive strategies, both immediately and after aging

Tested universal adhesive	Adhesive strategy	Immediate	Aged
Tetric Uni	SE	96.08 ± 2.31^a^	91.13 ± 3.32^b^
	ER 15s	98.02 ± 1.65^a^	90.27 ± 3.23^b^
	ER 3s	97.18 ± 1.33^a^	92.04 ± 5^b^
Prime&Bond Uni	SE	95.03 ± 1.80^a^	88.9 ± 3.18^b^
	ER 15s	97.14 ± 2.43^a^	91.08 ± 2.82^b^
	ER 3s	98.09 ± 1.80^a^	90.48 ± 2.02^b^

Abbreviations: SE: Self-etch; ER 15s, Etch and rinse with 15 s phosphoric acid etching; ER 3s, Etch and rinse with 3 s phosphoric acid etching. The statistical differences between the two aging conditions within each tested subgroup were assessed using a paired sample t-test (α = 0.05). Groups identified with the same superscripted lower case letters are not significantly different from each other

**Fig. 2:**
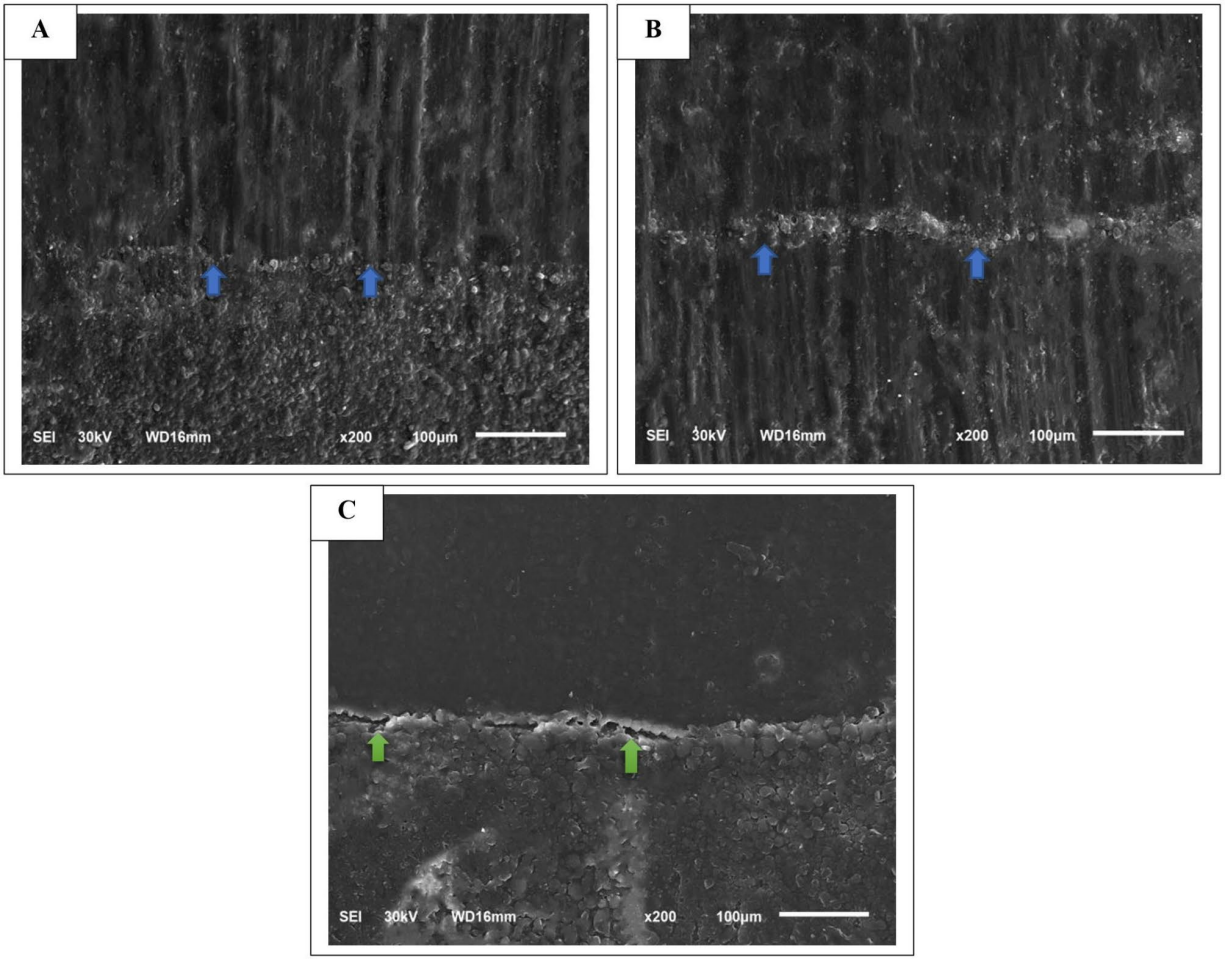
Representative SEM images of marginal adaptation evaluation at 200× magnification. Blue arrows in **A** and **B** indicate the resin composite/gingival dentin interface, highlighting the continuous margin, while green arrows in **C** point to a gapped margin

When comparing the immediate % CM values to the aged values, immediate measurements were higher across all subgroups. Further statistical examination using paired-sample t-tests revealed significant differences between immediate and aged values for all subgroups (*p* < 0.05) (Table 5). Representative SEM images for marginal adaptation evaluations, depicting both continuous and discontinuous margins, are shown in Fig. 2. The Pearson correlation coefficient between μTBS and % CM values revealed a moderately positive significant relationship (ρ = 0.472, *p* < 0.001).

**Table 5:** Results of comparing % CM values of marginal adaptation of tested universal adhesives using different adhesive strategies, immediately and after aging

	Paired differences					t	df	Sig. (2-tailed)
	Mean	Std. deviation	Std. error mean	95% Confidence interval of the difference				
				Lower	Upper			
Tetric Uni SE Immediate – aged	4.91	4.27	1.62	0.99	8.90	3.07	6	0.02
Tetric Uni ER 15s Immediate – aged	7.75	3.36	1.27	4.64	10.86	6.09	6	0.001
Tetric Uni ER 3s Immediate – aged	5.14	5.12	1.94	0.41	9.89	2.66	6	0.04
Prime&Bond Uni SE Immediate – aged	6.04	3.68	1.39	2.63	9.44	4.33	6	0.005
Prime&Bond Uni ER 15s Immediate – aged	6.06	4.34	1.64	2.04	10.08	3.69	6	0.01
Prime&Bond Uni ER 3s Immediate – aged	7.61	2.97	1.12	4.87	10.36	6.78	6	0.001

Abbreviations: SE: Self-etch; ER 15s, Etch and rinse with 15 s phosphoric acid etching; ER 3s, Etch and rinse with 3 s phosphoric acid etching

### Dentin etching patterns evaluation

**Fig. 3:**
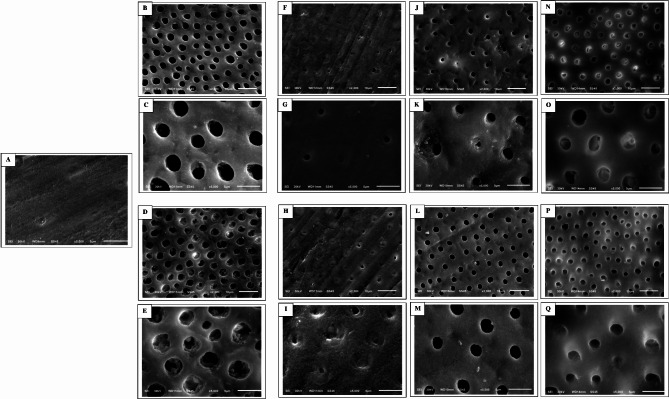
**A**, the non-etched dentin surface (at 5,000× magnification). **B** and **C**, dentin etching for 15 s (at 2,000× and 5,000× magnification, respectively). **D** and **E**, dentin etching for 3 s (at 2,000× and 5,000× magnification, respectively). **F**-**I**, the dentin surfaces treated with two universal adhesives in self-etch modes, **F** and **G** for Tetric Uni and **H** and **I** for Prime&Bond Uni at 2,000× and 5,000× magnification, respectively. **J**-**M**, the dentin surfaces treated with two universal adhesives in etch and rinse mode (15 s of etching), **J** and **K** for Tetric Uni and **L** and **M** for Prime&Bond Uni at 2,000× and 5,000× magnification, respectively. **N**-**Q**, the dentin surfaces treated with two universal adhesives in etch and rinse mode (3 s of etching), **N** and **O** for Tetric Uni and **P** and **Q** for Prime&Bond Uni at 2,000× and 5,000× magnification, respectively

The non-etched dentin surfaces displayed a dense smear layer covering the orifices of dentin tubules (Fig. 3, A). Dentin etching for 15 s resulted in the complete removal of both the smear layer and smear plugs (Fig. 3, B and **C**). However, when dentin was etched for only 3 s, the smear layer was only partially dissolved, leaving some residual smear plugs (Fig. 3, D and **E**).

When examining dentin surfaces treated with the two tested universal adhesives in self-etch mode, a residual smear layer and smear plugs remained (Fig. 3, F-I), indicating that both mild adhesives were unable to fully dissolve the smear layer in self-etch mode. In contrast, using both universal adhesives in etch and rinse mode with a 15-second etching time almost completely eliminated the smear layer and smear plugs (Fig. 3, J-M). When the adhesives were used in etch and rinse mode with a 3-second etching time, the smear layer was dissolved, but some smear plugs remained (Fig. 3, N-Q).

## Discussion

Hydroxyethyl methacrylate (HEMA) is a hydrophilic monomer that enhances the wetting properties of dental adhesives on dental surfaces [29]. However, its hydrophilicity makes it prone to hydrolysis and water absorption [29]. HEMA in universal adhesives can negatively interact with MDP, reducing demineralization and impairing the chemical bond between MDP and dental surfaces [30].

Cochinski et al. [31] found that two HEMA-free universal adhesives had lower bonding performance than a single HEMA-free adhesive, which showed bonding properties similar to a control with 10-MDP and HEMA. This suggests that adhesive characteristics, such as pH and solvent composition, influence bonding performance. Additionally, a study found that the same HEMA-free adhesive tested in the present study showed better bond strength with the etch-and-rinse technique compared to self-etch, indicating it may benefit from short dentin etching [32].

In this study, the etch-and-rinse strategy was performed using the air-drying technique, as recommended by the manufacturer of Tetric Uni. For Prime&Bond Uni, both air and blot drying were suggested, but only air drying was used to ensure standardization.

Restorative system adaptation is influenced by factors like resin composite shrinkage, which causes stress and marginal gaps [33]. Cavity size, type, and layering technique were standardized to account for shrinkage in the marginal adaptation test.

The study found that while the type of universal adhesive and adhesive strategy influenced bond strength, they did not affect adaptation, partially rejecting the first and second null hypotheses.

In contrast to Prime&Bond Uni, the bond strength of Tetric Uni was unaffected by the adhesive strategy used. Etching dentin removes the smear layer and exposes collagen for resin impregnation, but also depletes calcium phosphate, which can hinder chemical bonding [3]. In the etch-and-rinse mode, the primary bonding mechanisms are resin diffusion and hybridization [34]. The self-etch mode, however, uses acidic monomers to condition dentin without depleting calcium phosphate, enhancing chemical bonding with MDP monomers [13]. Research suggests that the bonding of mild universal adhesives to dentin is consistent across strategies [3], which may explain the lack of impact of adhesive strategy on Tetric Uni’s bond strength.

Despite containing 10-MDP and PENTA monomers, Prime&Bond Uni exhibited the weakest bond strength in self-etch mode. 10-MDP forms a stable ionic bond with dentin calcium, improving adhesion [35], but PENTA’s erythritol phosphate group may hinder calcium bonding due to steric effects, despite strengthening the polymer network. The poor performance in self-etch mode could also be due to the isopropanol solvent, which has a lower dielectric constant than ethanol. This can increase the pKa of acidic monomers, reducing hydrolyzed species and impairing calcium interaction [36]. In contrast to the results of the current study, Hardan et al. [13] reported comparable bond strength values for the same tested HEMA-free adhesive of the current study when used in self-etch mode and after phosphoric acid etching for 3 s. The difference could be explained by various factors in experimental design, including the type of teeth used; Hardan et al. [13] utilized bovine incisors. Additionally, the substrates for bonding were different, not only in terms of the type of teeth but also in that they bonded to the buccal dentin. Furthermore, the aging process differed from that of the current study. All of these factors could account for the disparities in results between the two studies.

The study found that aging significantly impacted bond strength and marginal adaptation, rejecting the third hypothesis. This decline is attributed to hybrid layer deterioration and mechanical stress from the mismatch in thermal expansion between tooth and restoration [37]. Despite differing compositions, all universal adhesives contained water, which can lead to hydrolysis of polymeric resins and enzymatic degradation of collagen fibrils after evaporation [38]. Simplified hydrophilic adhesives act as semi-permeable membranes, promoting water absorption and accelerating interface degradation and hydrolysis [39]. Repeated load cycling and temperature changes can cause micro-separations between the dentin surface and bonding agent, or plastic deformation of the adhesive interface [40]. Stress from these changes is concentrated at the interface between the bonding agent and the top of the hybrid layer, with occasional fractures at the bottom.

Both adhesives contain 10-MDP, which forms water-insoluble calcium salts. These salts may not immediately affect bond strength on self-etched dentin but are believed to protect the hybrid layer from hydrolytic degradation over time [39]. However, when 10-MDP is mixed with other resin monomers in dentin adhesives, its ability to enhance bond stability is uncertain due to hydrolytic degradation of the ester component and other methacrylate monomers [1]. Tetric Uni contains HEMA, which may compete with 10-MDP for calcium binding sites on apatite crystals, potentially weakening the chemical bond [30].

Enzymatic activity plays a significant role in bond degradation. Even adhesives without a separate etching step fail to prevent the activation of dentin matrix metalloproteinases (MMPs) [41]. Self-etch adhesives can expose and activate latent cysteine MMPs, increasing enzymatic activity [41]. This suggests that adhesives used in either etch-and-rinse or self-etch modes may impact bond durability due to proteolytic dentin activity.

Prime&Bond Uni exhibited lower bond strength than Tetric Uni after aging, regardless of adhesive strategy. Prime&Bond Uni lacks HEMA, which improves dentin wetting [42]. Without HEMA, adhesives can phase-separate when exposed to water, causing nano-leakage in the polymerized adhesive layer [43]. Tetric Uni’s HEMA and ethanol enhance wetting, reducing thickness and viscosity to maintain expanded collagen fibrils after solvent evaporation, improving monomer penetration into dentin. The etch-and-rinse strategy used in the study with air drying can collapse collagen fibrils, hindering adhesive diffusion [44]. Prime&Bond Uni’s solvent, isopropanol, has a lower hydrogen bonding capacity than ethanol, making it less effective at breaking interpeptide hydrogen bonds that stabilize the matrix and fibrils. Isopropanol also has a lower stiffening rate than ethanol, potentially increasing matrix shrinkage and reducing resin infiltration [45]. This could explain the significant drop in bond strength for Prime&Bond Uni compared to Tetric Uni after aging.

The study found similar bond strength and marginal adaptation for both etching durations in the etch-and-rinse strategy for both adhesives. Previous studies suggested that 3-second H_3_PO_4_ etching could enhance bonding [2, 12], but this was not observed here, even after aging. Failure modes were only influenced by aging, and while some studies link bond strength to failure mode [46], others do not [47]. Pre-test failure distribution was unaffected by study variables, suggesting random preparation issues [48]. A significant, moderately positive correlation was found between bond strength and adaptation values in every subgroup, leading to the rejection of the fourth null hypothesis. Contrary to the previous findings, a recent study reported that there was no significant correlation between μTBS and in vitro marginal gap formation in dentin [49]; thus, further investigation is needed.

The dentin-etching patterns observed at the interfaces of the tested bonds show that the mild universal adhesives have limitations in penetrating the smear layer created by the 600-grit silicon carbide paper. Unetched remnants of the smear layer were found after applying the adhesives in the self-etch mode for 20 s, as recommended by the manufacturer. In contrast, etching for 15 s completely removed the smear layer, and a 3-second H_3_PO_4_ etching also effectively eliminated most of it. While variations in dentin etching patterns and smear layer presence were noted across adhesive strategies, the impact of the adhesive strategy on bond strength depended on the adhesive composition. Despite differences in smear layer removal, no significant effect was observed on marginal adaptation for any of the adhesives. These findings highlight the need for further investigation.

The study had limitations. First, applying phosphoric acid for only 3 s in larger cavities may be impractical. Second, the limited range of universal adhesives tested affects the generalizability of the results. Future research should explore etching times longer than 3 s but less than 15 s, as well as investigate enzymatic activity in dentin following various etching durations. Additionally, comparing this activity across different universal adhesives in the self-etch mode would be valuable.

## Conclusions

1. The choice of adhesive strategy significantly influenced the dentin bond strength of the HEMA-free, isopropanol-based universal adhesive, with etch and rinse demonstrating superior performance over self-etch.
2. Bonding strategies exhibited consistent gingival margin integrity, regardless of aging.
3. Aging compromised both the bond strength and adaptation of the tested universal adhesives.
4. Three and 15-second etching times yielded comparable results, with extended H_3_PO_4_ application providing no additional advantages.

## Data Availability

The datasets generated and/or analyzed during the current study are not publicly available but are available from the corresponding author upon reasonable request.
